# Microbiological Quality and Presence of Foodborne Pathogens in Raw and Extruded Canine Diets and Canine Fecal Samples

**DOI:** 10.3389/fvets.2022.799710

**Published:** 2022-07-18

**Authors:** Doina Solís, Magaly Toro, Paola Navarrete, Patricio Faúndez, Angélica Reyes-Jara

**Affiliations:** ^1^Laboratorio de Microbiología y Probióticos, Instituto de Nutrición y Tecnología de los Alimentos (INTA), Universidad de Chile, Santiago, Chile; ^2^Red de Atención Veterinaria, Hospital de Alta Complejidad Bilbao, Universidad de Chile, Santiago, Chile

**Keywords:** raw meat-based diets, foodborne pathogens, pet food, pet food safety, *Listeria monocytogenes*, *Salmonella* spp., *Campylobacter* spp.

## Abstract

Pet food can be a source of microbiological hazards that might affect companion animals and owners. Even though owners usually rely on conventional pet diets, such as extruded diets, new feeding practices, such as raw meat-based diets (RMBDs), have grown. RMBDs' benefits are still scientifically uncertain, while its risks have been documented. The use of canine RMBDs might increase the exposure to zoonotic pathogens, such as *Salmonella* spp., *Listeria monocytogenes, Campylobacter* spp., among others. Identifying pathogen prevalence in canine food and pets is required to contribute to public health measures. The aims of this study were: (1) to compare the microbiological quality of RMBDs and extruded diets (2) to identify and compare the prevalence of *Salmonella spp., Campylobacter jejuni*, and *L. monocytogenes* from raw and extruded canine diets and canine fecal samples, and (3) to characterize pet owners according to the diet chosen to be used on their pets, their motivations for using RMBDs, and their knowledge about benefits and risks related to this feeding practice. Conventional and molecular microbiological methods were used to identify pathogen presence from food and fecal samples, while pulsed-field gel electrophoresis (PFGE) was performed to evaluate the clonal relationship between isolates. Aerobic plate counts for RMBDs were higher than those detected for extruded diets. *Salmonella* spp. and *L. monocytogenes* were isolated from 35.7% (15/42) RMBDs, while *Salmonella* spp., *C. jejuni*, and *L. monocytogenes* from 33.3% (11/33) fecal samples from RMBD-fed dogs. From the RMBD samples positive to *Salmonella* spp., chicken was the main meat ingredient composing the diets. PFGE analysis confirmed a genetic association between *Salmonella* spp. isolates from fecal and raw food samples from the same household. We did not detect pathogens from extruded food samples or feces from extruded-fed dogs. Using a survey, we identified dog owners' unawareness and/or underestimation of risks related to RMBDs. We demonstrated that canine raw pet food might be a source of zoonotic foodborne pathogens that represent a health risk for both humans and pets. While clinical findings caused by the mentioned pathogens vary among pets, the zoonotic potential implies a significant concern.

## Introduction

It is estimated that around 470 million dogs and 370 million cats are owned and kept as pets globally (1). The constant growth of the pet population and the close bond between humans and pets influence a high demand for high-quality pet products. In pet foods, safety is one of the most critical characteristics that must be assured along the production chain (2).

Since the mid-1,800 s, when the first commercial pet foods appeared, the pet food industry has continuously grown. By 2005 and subsequent years, dog's and cat's nutrition mainly consisted of conventional commercial diets, such as dry, moist, and semi-moist food (3). These diets are produced mostly through extrusion processes and have been historically considered safe to feed pets (4, 5).

Food safety threats that might be present through the pet food manufacturing process include physical, chemical, and biological hazards that are also present in the food industry intended for human consumption (6). Leading causes of pet food recall include mycotoxins, *Salmonella* spp., contamination with veterinary drugs, ingredient adulteration, and errors in the nutritional formulation (5, 6). The most significant sources of contamination are raw materials (6). Although extrusion is considered an effective critical control point (CCP) to eliminate microbiological hazards–such as *Salmonella* spp.—throughout the conventional production process, post-extrusion contamination and pet illnesses are still possible (5–7).

The close interaction between humans and pets has promoted companion animal lifestyle changes, including different dietary choices related to the owner's interests and beliefs (8). Although conventional commercial diets are still the preferred type of pet food chosen among pet owners, the interest in new feeding practices, such as the use of raw meat-based diets (RMBDs), has grown (4). Proponents believe this type of food is more natural and healthier for pets (9).

RMBDs consist of uncooked ingredients, including meats, organs, meaty bones, vegetables, and fruits (7, 10). Some of these ingredients, especially those of animal origin, are frequently contaminated with foodborne pathogens such as *Salmonella* spp. *L. monocytogenes, Campylobacter* spp., Shiga-toxigenic *Escherichia coli*, among others (9–11). Pathogen detection in RMBDs has been studied in countries where this feeding practice has been established for at least 30 years (11). The prevalence of these pathogens in RMBDs varies among countries, ranging between 7.1 and 80% for *Salmonella* spp. (11, 12), 16–54% for *L. monocytogenes* (10, 13), and 0–22% for *C. jejuni* (11).

Pets might also get ill due to pathogen infection, and raw diets have been recognized as a risk factor for fecal pathogen shedding in pets (9, 11). Moreover, contact with ingredients contaminated with antimicrobial-resistant bacteria might also be a public health concern for both the owner and the pet (4, 9). To date, most microbiological hazards present in the pet food industry still need effective control measures (2).

The present study sought to determine pet food safety based on the presence of zoonotic pathogens in extruded and RMBDs and to determine the prevalence of *Salmonella* spp., *C. jejuni*, and *L. monocytogenes* in canine fecal samples. Moreover, the genetic relationship between bacteria isolated from pet food and fecal bacterial isolates was studied. Additionally, to understand the factors that determined the adoption of the raw diets feeding practice, we examined the owners' motivations for feeding their dogs these diets. Finally, we characterized some in-home hygiene practices adopted by owners while manipulating RMBDs.

## Materials and Methods

### Pet Food Samples

A total of 66 dog food samples (RMBDs = 42; extruded diets = 24) were analyzed. RMBDs were divided into raw commercial (*n* = 31) and home-prepared raw diets (*n* = 11). Canine raw commercial diets were obtained from every participant or purchased by the investigators. All homemade RMBDs analyzed were obtained from owners. Commercial RMBDs came from the six most representative RMBD producers in the market, and selected companies were legally registered by the country's veterinary and health authorities (14). The numbers of samples from each manufacturer were: A = 5, B = 4, C = 5, D = 5, E = 6, F = 6. RMBDs were transported in coolers containing ice packs to the laboratory as previously described (9). Extruded diets (*n* = 24) were received from participants in sterile containers and transported at room temperature for further analysis.

### Fecal Samples

Pet owners were recruited through a public announcement emphasizing on RMBD-fed dogs. Upon the owner's acceptance of the terms of the study, a physical examination was performed by veterinarians. Dogs enrolled in the study were healthy, did not have any gastrointestinal symptoms, and did not use antibiotics for at least 6 weeks before the sample collection. The demographics of dogs enrolled in the study are shown in Supplementary Table 1. Rectal swabs (Copan^®^ Transystem™ 132C) were obtained from 33 RMBD-fed dogs and 22 extruded-fed dogs; every dog was sampled once, except one dog (T20–40), which was sampled on two different occasions. Samples were transported in coolers containing ice packs to the laboratory and processed within the same day. The study was approved by the Institutional Committee for the Care and Use of Animals (CICUA) from the University of Chile under the CICUA code 20411-VET-UCH.

### Microbiological Analysis of Pet Food Samples

All dog food samples (RMBDs = 42; extruded = 24; total = 66) were analyzed following methodologies described in the Bacteriological Analytical Manual (BAM) with minor modifications for aerobic plate counts (APC), *Salmonella* spp, *L. monocytogenes*, and *C. jejuni* isolation and identification (15–18).

For the APC analysis, 25 g from each sample were homogenized in 225 ml buffered peptone water (Bacto^TM^, 212367, Australia), and decimal dilutions were prepared. Afterward, 1 ml of each dilution was transferred into separate Petri dishes, and 15 ml of warm plate count agar was poured over the sample (Oxoid^TM^, CM0463, USA). Petri dishes were incubated at 37°C for 48 ± 2 h (18). An acceptable APC level (<1 × 10^6^ UFC/g) was defined according to the maximum level suggested by Kukier et al. (19).

*Salmonella* spp. isolation was conducted through a two-step enrichment procedure. Briefly, 25 g of each food sample were homogenized in 225 ml lactose broth (Difco^TM^, 241,000) and incubated at 37°C for 24 h. Then, 0.1 ml of the homogenate was inoculated onto Rappaport-Vassiliadis broth (Oxoid^TM^, CM0669, USA) and 1 ml onto tetrathionate broth (Oxoid^TM^, CM0671, USA). Both enrichments were incubated at 42°C for 24 h and then plated into Hektoen agar (BD Difco^TM^, 11703543, USA) and XLD agar (BD Difco^TM^, 11783503, USA) and incubated at 37°C for 24 h (15). Typical colonies were characterized by biochemical tests (15) and confirmed as *Salmonella* spp. through a polymerase chain reaction (PCR) for the *invA* gene with the primers *invA*F (5′-GAATCCTCAGTTTTTCAACGTTTC-3′) and *invAR* (5′- TAGCCGTAACAACCAATACAAATG-3′) (20).

For *L. monocytogenes* isolation, 25 g of sample were homogenized in 225 ml *Listeria* enrichment broth (BD Difco^TM^, 11718333, USA) and incubated at 30°C for 48 h. The enrichment was plated onto Palcam agar (Oxoid^TM^, CM0877, USA) and Chromogenic *Listeria* agar (Oxoid^TM^, CM1084, USA) and incubated at 37°C for 48 h (16). Presumptive *L. monocytogenes* colonies from each plate were confirmed by Gram stain observation and a PCR with the primers lmo3F (5′-GTCTTGCGCGTTAATCATTT-3′) and lmo4R (5′-ATTTGCTAAAGCGGGAATCT-3′) (21).

For *C. jejuni* isolation, food samples (25 g) were homogenized in 225 ml Bolton broth (Oxoid^TM^, CM0983, USA) with supplement SR0183 (Oxoid^TM^, USA) and 5% horse blood. Homogenates were incubated at 37°C for 24 h in microaerophilic conditions, followed by plating onto Skirrow agar (Oxoid^TM^, CM0331, USA) with supplement SR0069 (Oxoid^TM^, USA) and 5% horse blood and onto CCDA agar (Oxoid^TM^, CM0739, USA) with supplement SR0155 (Oxoid^TM^, USA). Plates were incubated at 42°C for 24−48 h in a microaerophilic atmosphere (17). Presumptive *C. jejuni* colonies were confirmed by microscopic observation (wet mount slides and Gram staining) and biochemical tests, including catalase and hippurate tests (17).

### Microbiological Analysis of Pet Fecal Samples

Canine fecal samples (RMBD-fed dog samples = 33; extruded-fed dog sample s = 22; total = 55) were also analyzed for the presence of the *Salmonella* spp., *L. monocytogenes*, and *C. jejuni*.

For *Salmonella* spp isolation, stool samples were inoculated into 10 ml buffered peptone water and incubated at 37°C for 24 h. Then, 0.1 ml were transferred to Rappaport-Vassiliadis and 1 ml to tetrathionate broth and incubated at 42°C for 24 h. Enrichments were plated onto Hektoen and XLD agar and incubated at 37°C for 24 h (15). Presumptive colonies were characterized by biochemical tests and confirmed as *Salmonella* spp. through a PCR for the *invA* gene as described in the previous section.

For *L. monocytogenes* isolation, stool samples were inoculated into 10 ml *Listeria* enrichment broth and incubated at 30°C for 48 h. Enrichment cultures were plated onto Palcam and Chromogenic *Listeria* agar and incubated at 37°C for 48 h (16, 22). Presumptive colonies were confirmed by PCR with primers lmo3F and lmo4R as described in the previous section.

For *C. jejuni* detection, fecal samples were inoculated into 10 ml Bolton broth with antibiotics and 5% horse blood and incubated at 37°C for 24 h in microaerophilic conditions. Enrichment cultures were plated onto Skirrow agar with supplement SR0069 and 5% horse blood and onto CCDA agar with supplement SR0155. Plates were incubated at 42°C for 24–48 h in a microaerophilic atmosphere and *C. jejuni* was identified as described in the previous section.

All bacterial isolates were stored at −20°C for further analysis.

### Pulse-Field Gel Electrophoresis

*Salmonella* spp. isolates (*n* = 10) obtained simultaneously from pet food and fecal samples from the same households were genetically characterized to assess their clonal relationship. Every isolate analyzed was obtained from a different dog, except two *Salmonella* spp. isolates (T20–40 A and B), obtained from fecal samples taken 1 month apart from the same dog. Pulsenet PFGE protocols were used (23). Briefly, isolated colonies were plated onto Trypticase Soy Agar (TSA) (BD Difco^TM^, 236950, USA) and incubated at 37°C for 14–18 h. Cell suspension plugs were prepared the next day in 1% Pulsed Field Certified Agarose (Bio-Rad, 1620137, USA) and transferred to lysis buffer (50 mM Tris:50 mM EDTA, pH 8.0 + 1% Sarcosyl) and Proteinase K (20 mg/ml). After washing the agarose plugs, digestion was performed with the *Xba*I enzyme (Promega, Wisconsin, USA) at room temperature for 15 min. The electrophoresis was performed on the CHEF DR III unit (Bio-Rad Laboratories Canada, Ltd., Mississauga, Ontario, Canada) using the conditions described in the *Salmonella* spp. protocol (initial switch time: 2.2 s, final switch time: 63.8 s, voltage:6 V, included angle:120°, run time: 17–20 h). BioNumerics version 7.1 (Applied Maths, Sint-Martens-Latem, Belgium) was used to interpret PFGE patterns and build a neighbor-joining (NJ) tree. Similarities of PFGE patterns were calculated using the Dice coefficient with a 1% tolerance.

### Owner's Motivations and Habits

A questionnaire to understand the owner's motivations and habits was developed by veterinarians and filled out by every owner. Surveys consisted of close-ended questions including three categories: (i) animal and diet-specific factors, (ii) owners' motivations for feeding the type of diet they had selected for their pets, and (iii) food hygiene practices. Open-ended questions were used to describe the owner's knowledge about risks and benefits related to raw feeding. The questionnaire for RMBD-fed dog owners (*n* = 22) included 22 questions, while only 14 questions were included for the extruded-fed group (*n* = 14). Extra questions answered by RMBD-fed dog owners consisted of the main raw ingredients used to feed their pets. Some participants had more than one dog, but those owners filled a single questionnaire.

### Statistical Analysis

Aerobic plate count data for RMBDs and extruded diets were tested for normality (Shapiro-Wilk test) (24) and homoscedasticity (Fligner-Killeen test) (25). Welch's two-sample *t*-test was used to contrast raw and extruded diets APC levels (26), while ANOVA was used for the multiple comparison analysis among RMBDs producers (27). Fischer's exact test was used to evaluate the association between pathogen prevalence and type of diet (RMBDs or extruded) (28). The Kruskal Wallis test was applied to analyze associations between a) pathogen presence and APC levels and b) pathogen presence and type of meat used in raw diets (29). A *p*-value of ≤ 0.05 was considered significant. Analyses were performed with the statistical software STATA (STATA^®^ MP 16.0, StataCorp, USA). Descriptive statistics were employed for the questionnaire's categorical variables, such as participants' gender/sex, age, and type of food used/consumed (30).

## Results

### Pet Food Microbiological Analysis

**Figure 1:**
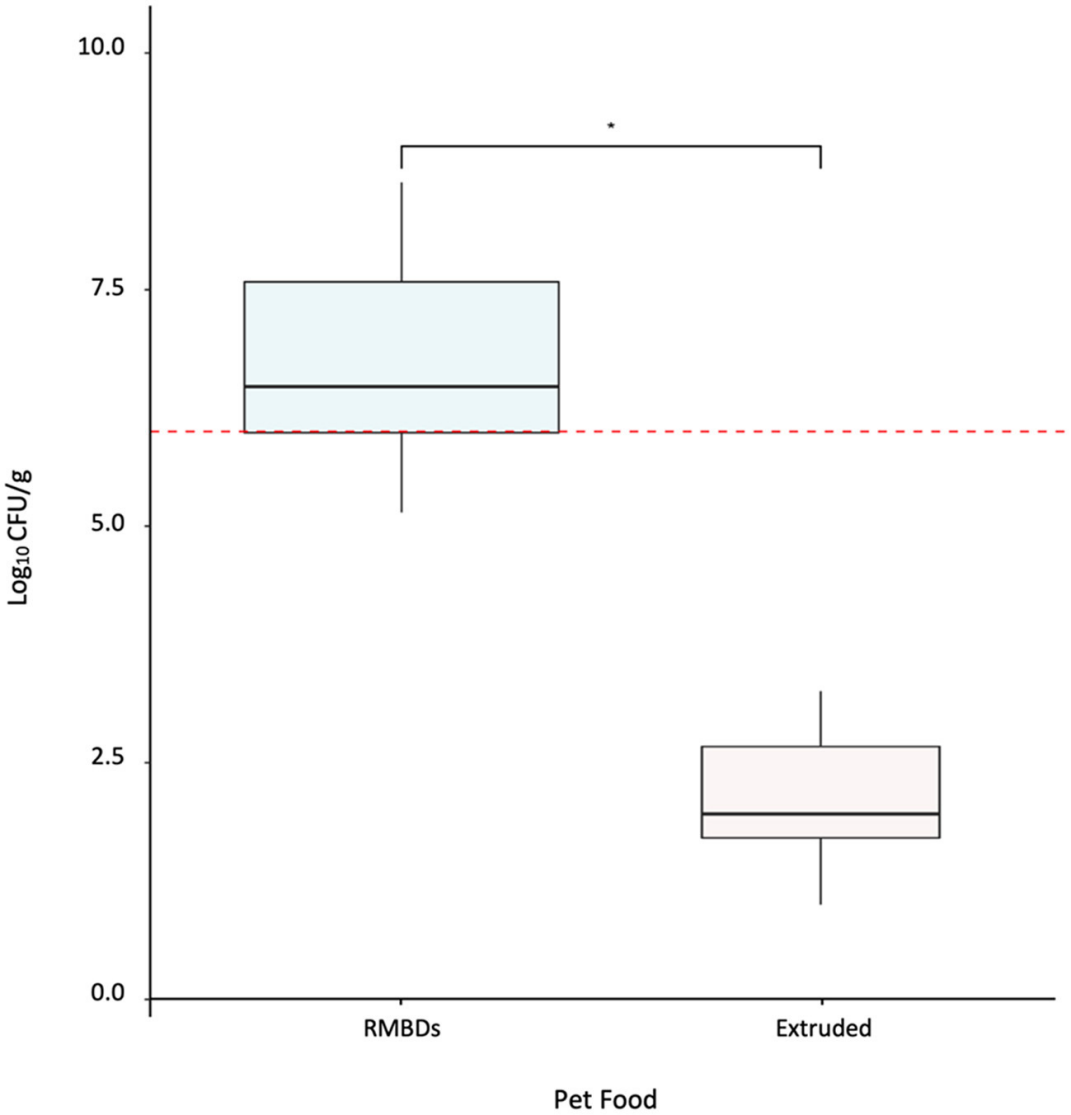
Aerobic bacterial counts (APC) for RMBDs (*n* = 42) and extruded (*n* = 24). The Fligner-Killeen test was used to determine the homogeneity of variances (homoscedasticity) and Shapiro-Wilk test for normality. The level of APC between both diets was compared using *Welch's Two-Sample T-test* *p-value < 2.20 × 10^−16^. The red line shows the suggested APC upper limit for animal compound feed according to Kukier et al. (19). RMBD's APC >1 × 10^6^ CFU/g = 31.

**Figure 2:**
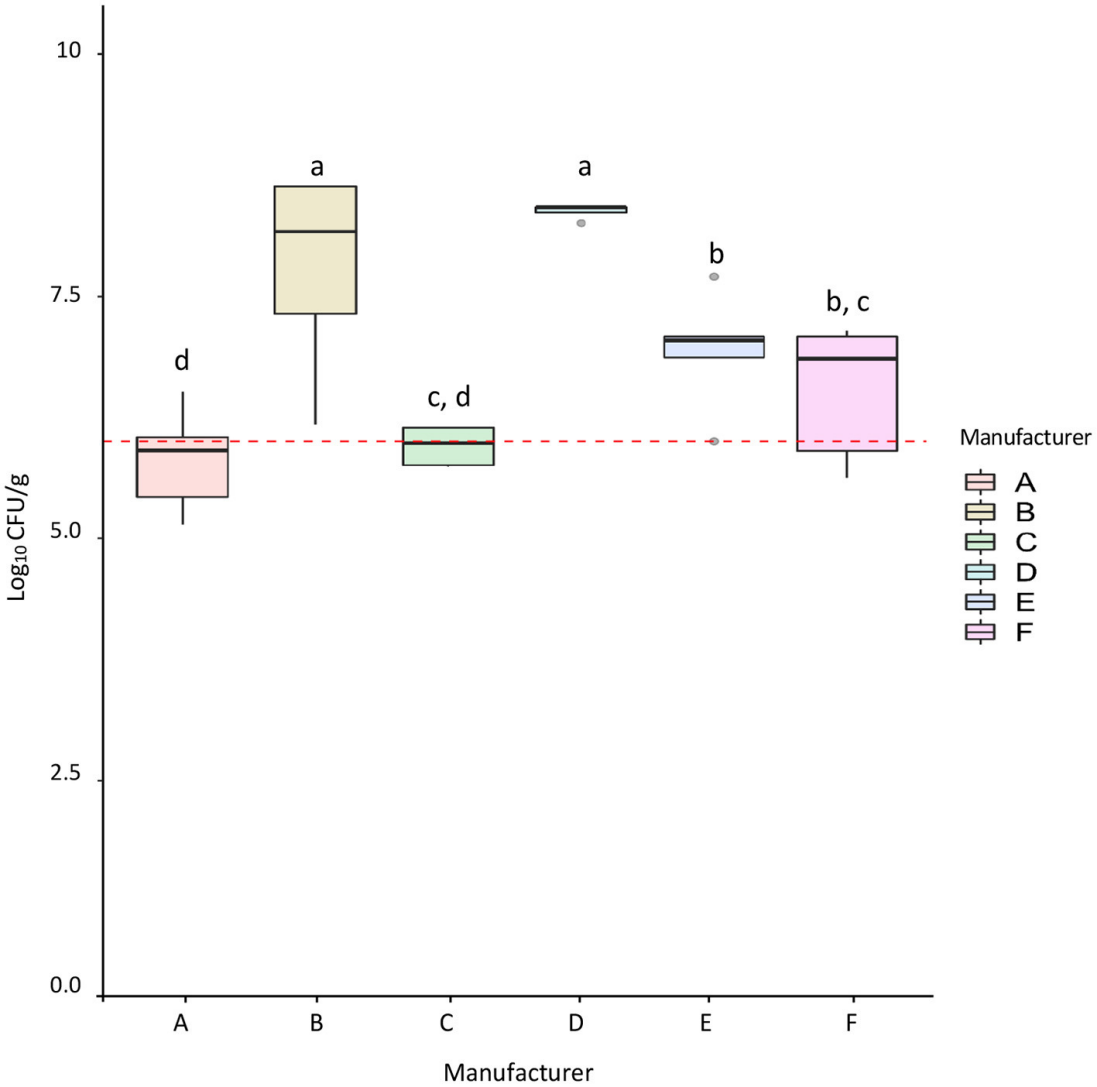
Aerobic bacterial counts (APC) for commercial RMBDs from different manufacturers. The number of samples from each manufacturer was: A = 5, B = 4, C = 5, D = 5, E = 6, F = 6. The Fligner-Killeen test was used to determine the homogeneity of variances and the Shapiro-Wilk test for normality. Multiple comparison analysis of ANOVA for the APC was performed between manufacturers. Different letters marked for bars represent significant differences (*p*-value < 0.05). The red line shows the suggested APC upper limit for animal compound feed according to Kukier et al. (19).

RMBDs (*n* = 42) and extruded (*n* = 24) pet food samples were analyzed using conventional and molecular microbiological methods to compare APC levels and pathogen presence. APC levels for the RMBDs, including commercially and homemade diets, were significantly higher than those detected for extruded diets (*p*-value <2.2 × 10^−16^) (Figure 1). For the RMBDs, APCs ranged from 1.4 × 10^5^ to 4.3 × 10^8^ CFU/g (Median = 3.0 × 10^6^), while for extruded diets it fluctuated from < 10 to 1.8 × 10^3^ CFU/g (Median = 9.1 × 10^1^) (Figure 1). No statistical difference was detected between commercial and homemade RMBD APCs (*p*-value = 0.17) (Supplementary Figure 1). Furthermore, from the six commercial RMBD manufacturers (A–F) analyzed, B and D showed the highest APCs (*p*-value < 0.05) (Figure 2). Statistical analysis showed that APCs were independent of the type of meat used to manufacture the RMBDs (*p*-value = 0.063, Kruskal Wallis).

None of the pathogens were isolated from the extruded diets, whereas 15/42 (35.7%) RMBD samples—commercial and homemade— carried bacterial pathogens. *Salmonella* spp. was isolated from 26.2% (*n* = 11) RMBD samples and *L. monocytogenes* from 19% (*n* = 8). *C. jejuni* was not isolated from any food sample. Chicken meat was the main ingredient in most of the food samples contaminated with *Salmonella* spp. (6/11; 54.5%) (Supplementary Table 2). *L. monocytogenes* was isolated from samples made with chicken (*n* = 2), beef (*n* = 2), salmon (*n* = 1), and guanaco (*Lama guanicoe*; *n* = 1) (Supplementary Table 2).

### Pet Fecal Samples Microbiological Analysis

A total of 55 fecal samples (RMBD-fed dog samples = 33; extruded-fed dog samples = 22) were analyzed for the presence of selected pathogens. We detected a higher prevalence of pathogen shedding from RMBD-fed dogs (33.3%; 11/33) than from extruded-fed dogs, in which samples did not carry any of the tested pathogens (0%; 0/22) (*p*-value = 0.002) (Table 1). Among the RMBD-fed dogs, *Salmonella* spp. was the pathogen most frequently isolated (24.2%, 8/33), while *L. monocytogenes* was isolated from 3% (1/33) and *C*. *jejuni* from 6% (2/33) fecal samples (Table 1).

**Table 1:** Total fecal samples analyzed (*n* = 55) and number of positive samples for *Salmonella* spp., *L. monocytogenes, and C. jejuni* according to the type of pet food.

**Type of pet food**	**Fecal samples n**	**Fecal samples with pathogens *n* (%)**	***Salmonella* spp. *n* (%)**	***L. monocytogenes n* (%)**	***C. jejuni n* (%)**
RMBDs	33	11 (33.3%)*	8 (24.2%)	1 (3%)	2 (6%)
Extruded	22	0 (0%)*	0 (0%)	0 (0%)	0 (0%)

*Fisher's Exact Test (p-value = 0.002).

### Association Between Bacterial Pathogen Shedding and Pet Food Contamination

An additional analysis was performed to evaluate the pathogen co-occurrence between isolates from fecal and food samples. Only bacterial contaminated pet food consumed by dog participants (*n* = 11) and canine pathogen carriers (*n* = 11) were considered for this analysis (Table 2).

**Table 2:** Analysis of the co-occurrence between isolates from pet food consumed by dog participants (*n* = 11) and the pathogen dog carriers (*n* = 11).

		**Fecal samples**			**RMBD samples**		
**Dog no**.	**Household**	***Salmonella* spp**.	***C. jejuni***	***L. monocytogenes***	***Salmonella* spp**.	***C. jejuni***	***L. monocytogenes***
5	a		x				
8	b						x
9	b						
11	c	x					
13	c	x					
14	d				x		
15	e				x		x
17	f				x		x
25	g	x			x		
28	h	x					
32	i		x		x		x
34	j	x			x		
40	k	x*			x		
45	l	x			x		
46	l	x			x		
52	m			x			

*Dog no. 40 was sampled twice. Both analyzes were positive for Salmonella spp. Pathogen co-occurrence (fecal and RMBD samples) is shown in gray.

Fecal samples from five dogs tested positive for pathogens, but their food tested negative (Table 2). Similarly, pathogens were detected in five RMBD samples obtained from households where dogs' fecal results did not evidence pathogen presence. However, in five cases, *Salmonella spp*. was obtained simultaneously from the pet fecal sampling and the dog's food. A dog fecal sample carried *C. jejuni*, but the pet consumed a RMBD contaminated with *Salmonella* spp. and *L. monocytogenes* (Table 2).

**Figure 3:**
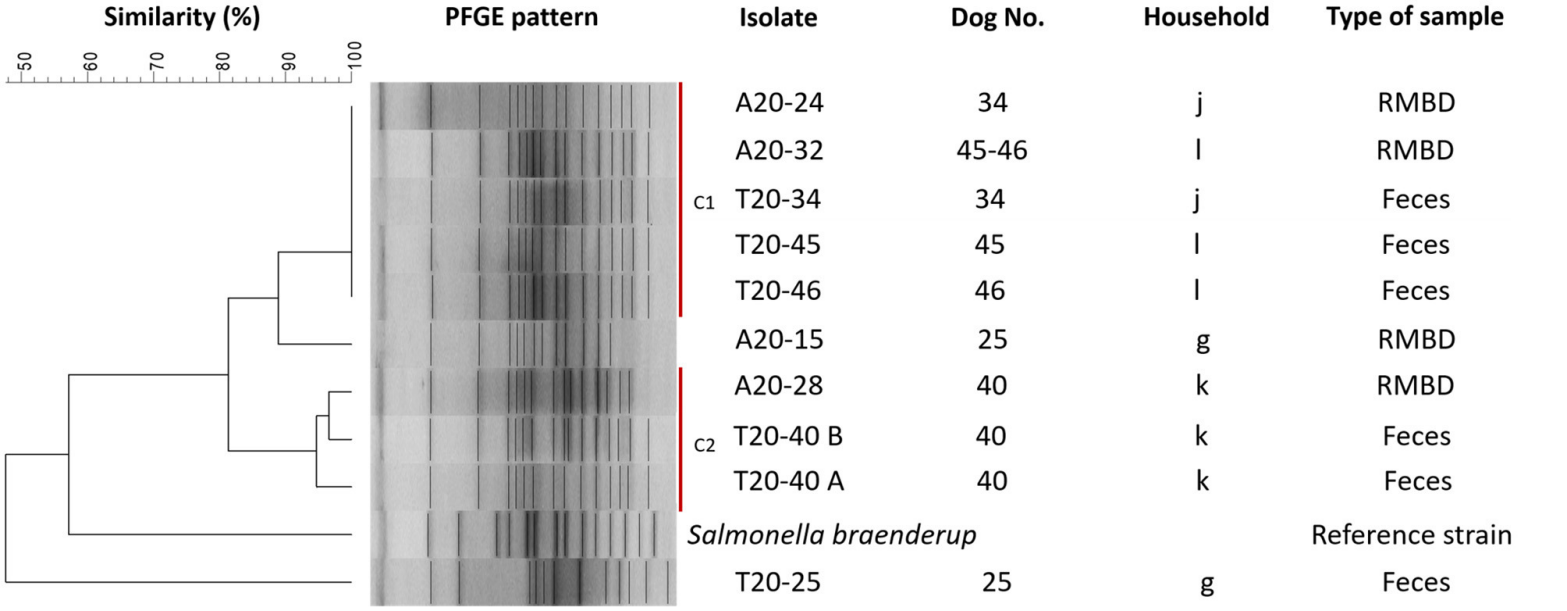
Dendrogram of pulsed-field gel electrophoresis (PFGE) cluster analysis of ten *Salmonella spp*. isolates from RMBD and fecal samples from RMBD-fed dogs. PFGE was performed for *Salmonella* spp. with the restriction enzyme *Xba*I. Sample T20–40 represents two different samples from the same patient taken 1 month apart. C1, cluster 1; C2, cluster 2.

*Salmonella* isolates (*n* = 10) obtained simultaneously from a dog's fecal sample and the RMBD the dog was fed with were analyzed by PFGE to assess their clonal relationship. The Neighbor-Joining tree, built with the restriction patterns resulting from the PFGE analysis, grouped isolates in two clusters with ≥90% similarity among isolates (Figure 3). Cluster 1 included five highly related *Salmonella* spp. isolates: food isolate A20–32 and fecal isolates T20–45 and T20–46 were obtained from “household l,” and food isolate A20–24 and fecal isolate T20–34 were associated with “household j.” Cluster 2 was formed by three *Salmonella* spp. from “household k:” A20–28 from pet food and two isolates (T20–40 A and B) from dog 40's fecal samples obtained 1 month apart (Figure 3). Interestingly, all these isolates came from households that used diets made with chicken as a primary meat ingredient.

### Characterization of Pet Owners

A total of 36 dog owners from 55 healthy dogs (RMBD-fed dogs = 33; extruded-fed dogs = 22) agreed to participate in the study and responded to the questionnaire. In some cases, participants owned more than one dog, which were fed the same type of diet. Since recruitment emphasized RMBD-fed dog owners, 61.1% (*n* = 22) of participants reported using RMBDs for their dogs, while 38.9% (*n* = 14) preferred conventional extruded diets. A summary of the most relevant demographic data from the respondents is shown in Supplementary Table 3.

The questions, mainly focusing on RMBD-fed dog owners, were intended to describe the owner's reasons and habits for using unconventional diets. Regarding the preferred ingredients for feeding their pets, the most common meat sources were beef and chicken, while liver was the main organ used. Apple and carrots were the most common fruit and vegetable used (Supplementary Figure 2).

The owners' perception of the risks and benefits of RMBDs was analyzed. All RMBD-fed dogs owners (*n* = 22) considered this feeding practice to provide numerous health benefits to their pets, mainly related to improved cutaneous and gastrointestinal health and better feces quality (Table 3; Supplementary Table 4). Nevertheless, only 12/22 (54.5%) declared knowing about the risks related to raw feeding. Moreover, 50% of extruded-fed dog owners knew about the risks and/or benefits of raw feeding (Table 3). Microbiological contamination of food and foreign bodies, such as bones, were the main risks perceived by the owners (Supplementary Table 4).

**Table 3:** Answers to questions to evaluate owners' knowledge about benefits (QA) and risks (QB) related to RMBDs.

**Variable**	**RMBD-fed dog owners (*n* = 22), *n* (%)**	Extruded-fed dog owners (*n* = 12*), *n* (%)
**QA: Knowledge about health benefits related to RMBDs**		
Yes	22 (100)	6 (50)
No	0 (0)	6 (50)
**QB: Knowledge about risks related to RMBDs**		
Yes	12 (54.5)	6 (50)
No	10 (45.5)	6 (50)

^*^Two owners refused to answer QA and QB.

RMBD-fed dog owners' habits showed that 68.2% (15/22) stored raw ingredients and prepared raw food in the same space/fridge used for human food storage. Also, 81.8% (18/22) of owners manipulated raw pet diets in the same space they prepared food for human consumption (Supplementary Table 5).

## Discussion

Pet ownership has grown throughout the years, and the human-animal bond has strengthened. Studies have shown that companion animals play an essential role in human health and wellbeing. Dog's role has diversified from simple companionship to new applications such as assistance and working and supporting humans in several therapeutic ways (31). Increasing pet ownership has also played an essential role in dogs' lifestyles, behaviors, and habits. Owners have changed their pet's feeding habits, including providing unconventional pet food like RMBDs (8). Raw diets might represent a source of microbiological hazards for pets, and these risks could even affect owners through contact with their pets or manipulation of contaminated pet food (8).

In this study, 31/42 raw diets analyzed showed an elevated APC level (Figure 1). Since Chilean regulation does not establish microbiological criteria exclusively for raw pet food (32), we defined an APC level of 10^6^ CFU/g as the maximum accepted level of microbial contamination as previously suggested by Kukier et al. (19). Since APC is a hygienic indicator used to estimate the bacterial population in a food sample (18), our results reveal the importance of strengthening good manufacturing practices in the RMBD industry. Overall, the microbiological quality for the extruded food in this study was adequate. Even though the microbiological quality of pet foods has been previously reported, only a few studies have compared the microbial load between raw and extruded diets (19), as shown in the present work.

Commercial RMBDs should be produced following strict safety measures (HACCP systems, handling of raw materials, hygienic and sanitary practices) and kept frozen; therefore, a lower microbiological load would be expected (33, 34). However, in the present study, we did not find significant differences in the APCs between homemade and commercial RMBDs (Supplementary Figure 1).

Pet diets are not exempt from microbiological hazards (5, 7). Even conventional diets that undergo an extrusion process might exhibit post extrusion contamination (5). From March 2020 to August 2021, the FDA started 28 recalls due to possible or confirmed microbiological contamination of pet food; 18 recalls for raw pet food, seven for extruded food, and three for pet treats. *Salmonella* spp., *L. monocytogenes*, Shiga toxin-producing *Escherichia coli* O128, and *Clostridium botulinum* were the cause of these recalls (35). Raw pet diets have shown to be a significant source of pathogenic bacteria (7, 9, 10). Our results support these findings since bacterial pathogens were isolated from 35.7% of the RMBD samples. We identified a similar detection rate for *Salmonella* spp. than other studies (10, 36, 37), and a lower detection rate for *L. monocytogenes* than previously reported (10).

In the present study, chicken was the primary source of animal protein in RMBDs contaminated with *Salmonella* spp, while diets contaminated with *L. monocytogenes* were made of diverse meat types (Supplementary Table 2). Chicken is a food matrix frequently linked to *Salmonella* spp. contamination (38, 39), while *L. monocytogenes* contamination has been linked to various food matrices, such as raw meat, fruits, and vegetables (40).

Worldwide, official microbial quality standards regulating raw pet foods are still limited (11), especially in regions where raw feeding is relatively new, such as Latin America. Even though regions such as the UK regulate the pet food industry, microbiological analysis for pet foods only consider *Enterobacteriaceae* counts and *Salmonella* spp. detection. Nevertheless, studying other possible microbial pathogens is supported by guidelines and recommendations that promote best practices throughout the pet food industry (41–43). For instance, the Pet Food Manufacturers Association (PFMA) guidelines for manufacturing raw pet food in the UK mention *L. monocytogenes, C. jejuni*, and *E. coli* as part of the microbiological hazards that should be considered in a risk assessment for raw pet foods. These pathogens are not yet legally regulated within the pet food industry but are closely related to human foodborne diseases (13, 40, 42, 44, 45). Including these foodborne pathogens in the regulation could reinforce the existing regulatory measures to safeguard pet and human health since these pathogens might cause severe human illness through direct contact with RMBDs or cross-contaminated surfaces while manipulating raw ingredients and products.

It should be considered that indicator microorganisms are not suitable as a direct assessment of safety, since the analyses does not differentiate between bacterial species. In the present study, we analyzed APCs instead of counting *Enterobacteriaceae*. APC is a quality indicator that estimates the number of total aerobic microorganisms that grow at mesophilic temperatures. It is used to evaluate the quality of raw materials, cross-contamination, and appropriate hygiene measures, among others (46). In the food industry, *Enterobacteriaceae* counts are used as an indicator of hygiene and post-processing contamination. This latter analysis is usually applied to heat-treated products, such as extruded pet diets, to assess the adequacy of processing and hygiene practices (46, 47). Although *Enterobacteriaceae* counts were not performed in this study, the resulting information could have been helpful as an indirect estimation of enteropathogen loads.

The information generated in this study supports the importance of implementing more robust measures, such as requiring HACCP implementation along the production chain and validating such systems. Moreover, sampling guidelines and microbiological criteria explicitly developed for RMBDs should be a requirement, and further studies will be necessary to identify other possible microbiological hazards in this type of product (9, 11).

The risk of pathogen shedding in pets has been previously studied. Authors have reported *Salmonella* fecal excretion in 3–50% of RMBD-fed dogs, and other pathogens have also been detected (7). For instance (48), in a recent study comparing fecal pathogen excretion between dry and raw-fed dogs, concluded that RMBD-fed dogs were 30 times more likely to excrete *Salmonella* than dogs fed with extruded diets (48). Nevertheless, a comparison of *L. monocytogenes* and *C. jejuni* shedding between raw and extruded-fed dogs has not been reported to date.

Reports indicate that *Salmonella* spp. is shed by 0–44% of dogs, and percentages vary among countries, feeding behaviors, and health status (48, 49). In our study, *Salmonella* spp. fecal excretion in RMBD-fed dogs (24.2%) was higher than in other existing reports (48) but inferior to data reported in Canada and other locations by Finley et al. (34, 50). Although *L. monocytogenes* was isolated in one fecal sample (3%), our study is one of the few available to report the canine fecal shedding of this pathogen. Moreover, our study is the first in comparing the fecal excretion of *L. monocytogenes* between raw and extruded-fed healthy dogs. Kocabiyik et al. (22) reported an *L. monocytogenes* fecal carriage of 1.22% in stray dogs; however, the study did not inform the type of food consumed by the dogs or whether a medical examination was performed on the dogs enrolled in the study before sampling.

The link between canine fecal pathogen shedding and consumption of contaminated RMBDs has been studied for *Salmonella* spp. (11, 48). Evidence for this link is limited for *C. jejuni* (11) and is not available for *L. monocytogenes*. In the present study, fecal isolation of *C. jejuni* and *L. monocytogenes* was independent of the detection of these pathogens in pet food samples.

Even though infections caused by *Salmonella, L. monocytogenes*, and *C. jejuni* are usually uncommon in dogs, cases have been strongly associated with ingesting contaminated meat or meat by-products (51). More importantly, the zoonotic potential, implicit public health risks (10), and severity of diseases caused by these pathogens represent the main risk of these types of pet food (52).

In this study, *C. jejuni* was detected only in 6% of stool samples, which is lower than previous findings (53). However, the pathogen was not detected in any of the food samples. Studies have shown an isolation rate of *C. jejuni* ranging from 0 to 50% in dogs with and without gastrointestinal disease (51). While the *Campylobacter* infection can be attributed to meat products, pathogen isolation from feces might not be simultaneous since the fecal shedding can last for prolonged periods after infection recovery (51). Moreover, viable non-culturable *Campylobacter* forms might also interfere with the isolation of the pathogen, even though bacteria in this form might still cause infection (54).

It has been described that RMBD-fed dogs can shed *Salmonella* shortly after the ingestion of contaminated pet food, and shedding might last up to 3–6 weeks in most cases. Usually, pathogen shedding is continual for the first week, but afterward, it becomes intermittent (34, 51). Dogs whose fecal sample tested positive for *Salmonella* spp., but the pathogen was not detected from their food were possibly sampled several days after ingesting contaminated raw food. Other factors might affect the bacterial detection from food samples, including the microbial distribution and the sample's heterogeneous structure, which might affect the sample representativeness and cause false-negative results (55, 56). Also, animal carriers might shed *Salmonella* due to an unrelated event, such as contact with contaminated fomites (food bowls, hospital cages), consumption of animals that act as reservoir hosts, coprophagy, among others (51). For dogs who showed a positive RMBD sample with a negative fecal result, the intermittency of the fecal shedding should also be considered, as well as the amount of ingested bacteria to produce gastrointestinal colonization and the pet's immune status, among other factors, since bacterial shedding and further bacterial isolation depends on the amount of bacteria that previously survived the passage through the stomach (51).

The genetic similarity among strains simultaneously isolated from dog fecal samples and RMBDs suggests the survival and spread of *Salmonella* spp. from the ingredients commonly used to prepare RMBDs (57). More dogs and a higher sampling frequency are required to perform a conclusive evaluation regarding clonal spread. In contrast, the possible clonal strains' spread among pets and owners might be assessed by studying both human and pet samples.

Evidence shows a high association between RMBDs and health hazards for both humans and animals (7, 9, 10); however, in this study, pet owners demonstrated a strong belief that RMBDs provide numerous benefits at minimal or no risks. These results are also in concordance with previously published data where authors reported owners' tendency to underestimate the risks related to raw feeding or their unawareness (30, 48). The questionnaire also revealed that owners' attitudes, practices, and motivations for using an unconventional raw diet were similar to those reported in other countries such as Brazil and Italy (30, 48).

Owners might be exposed to pathogens through contact with pet food, direct contact with the pet excretions, and the dog's environment. For example, cross-contamination while manipulating pet RMBDs is another critical factor that might cause human illness (9). It is crucial to promote the interdisciplinary work between health professionals and national and international agencies to provide guidance and work in safety strategies for minimizing the risks that might be present in pet products, especially in pet food. The One Health approach is mandatory to guarantee the optimum and healthy interconnection between humans and pets and the health of their shared environment (58).

## Conclusion

We concluded that RMBDs are more likely to be contaminated by pathogenic bacteria than extruded pet diets. Along the raw pet food manufacturing process, safety measures should be strengthened to control possible microbiological hazards that might affect dogs and owners, and implemented in countries where regulation is absent. Novel microbial inactivation technologies and strategies, or the improvement of already known technologies, such as high-pressure processing, should be studied to control contaminating pathogens. Enteropathogen excretion was higher in RMBD-fed dogs than in extruded-fed dogs. At the same time, the eventual spread of bacterial clones such as *Salmonella* spp. from RMBD to dogs or owners might represent a significant public health concern. Furthermore, owners who preferred raw diets for their pets underestimated the risks related to this feeding practice or knew the risks but still chose to use these diets. Education, guidance, and proper communication between health professionals and pet owners are required, especially for the owners who prefer raw diets for their pets, since the correct use of RMBDs might minimize the risk of food-borne contamination to both owners and pets. Since this is the first Chilean study addressing this topic, further research in this area, the analysis of different zoonotic pathogens, and a larger sample size will be a useful complement to the reported data.

## Data Availability Statement

The original contributions presented in the study are included in the article/Supplementary Material, further inquiries can be directed to the corresponding author/s.

## Ethics Statement

The animal study was reviewed and approved by Institutional Committee for the Care and Use of Animals (CICUA) from the University of Chile under the CICUA code 20411-VET-UCH. Written informed consent was obtained from the owners for the participation of their animals in this study.

## Author Contributions

DS and AR-J: conceptualization of the study and conceived the original idea. DS and PF: sample collection. DS: carried out the experiments. DS, MT, and AR-J: microbiological data analysis, application of statistical analysis, and contributed to the interpretation of the results. DS, MT, PN, PF, and AR-J: writing–reviewing and editing. All authors read and approved the final manuscript.

## Funding

The ENL12/20, a grant from University of Chile support the pay of supplies to perform the research.

## Conflict of Interest

The authors declare that the research was conducted in the absence of any commercial or financial relationships that could be construed as a potential conflict of interest.

## Publisher's Note

All claims expressed in this article are solely those of the authors and do not necessarily represent those of their affiliated organizations, or those of the publisher, the editors and the reviewers. Any product that may be evaluated in this article, or claim that may be made by its manufacturer, is not guaranteed or endorsed by the publisher.

## Supplementary Material

Supplementary Figure 1 Aerobic bacterial counts (APC) for commercial (*n* = 31) and homemade (*n* = 11) RMBDs. The Fligner-Killeen test was used to determine the homogeneity of variances and the Shapiro-Wilk test for normality. The level of APC between both diets was compared using *Welch's Two-Sample T-test, p*-value = 0.17. The red line shows the suggested APC upper limit for animal compound feed according to Kukier et al. (19).

Supplementary Figure 2 Most common raw ingredients preferred by pet owners for commercial and/or homemade RBMDs (*n* = 42). **(A)** Meat, **(B)** Organ meats, **(C)** Fruits, **(D)** Vegetables.

Supplementary Table 1 Demographics of dogs enrolled in the study (*n* = 55).

Supplementary Table 2 Characterization of the RMBDs analyzed (n = 42).

Supplementary Table 3 Demographics of dog owners (n = 36).

Supplementary Table 4 RMBD-fed dog owners' perceptions about risks and benefits related to RMBDs.

Supplementary Table 5 RMBD-fed dog owners' main characteristics and habits.
