# Supplementary figures and images for: Microbiological Quality and Presence of Foodborne Pathogens in Raw and Extruded Canine Diets and Canine Fecal Samples
Source: Front Vet Sci. 2022 Jul 18;9:799710. doi: 10.3389/fvets.2022.799710 (PMC9339799; doi:10.3389/fvets.2022.799710)

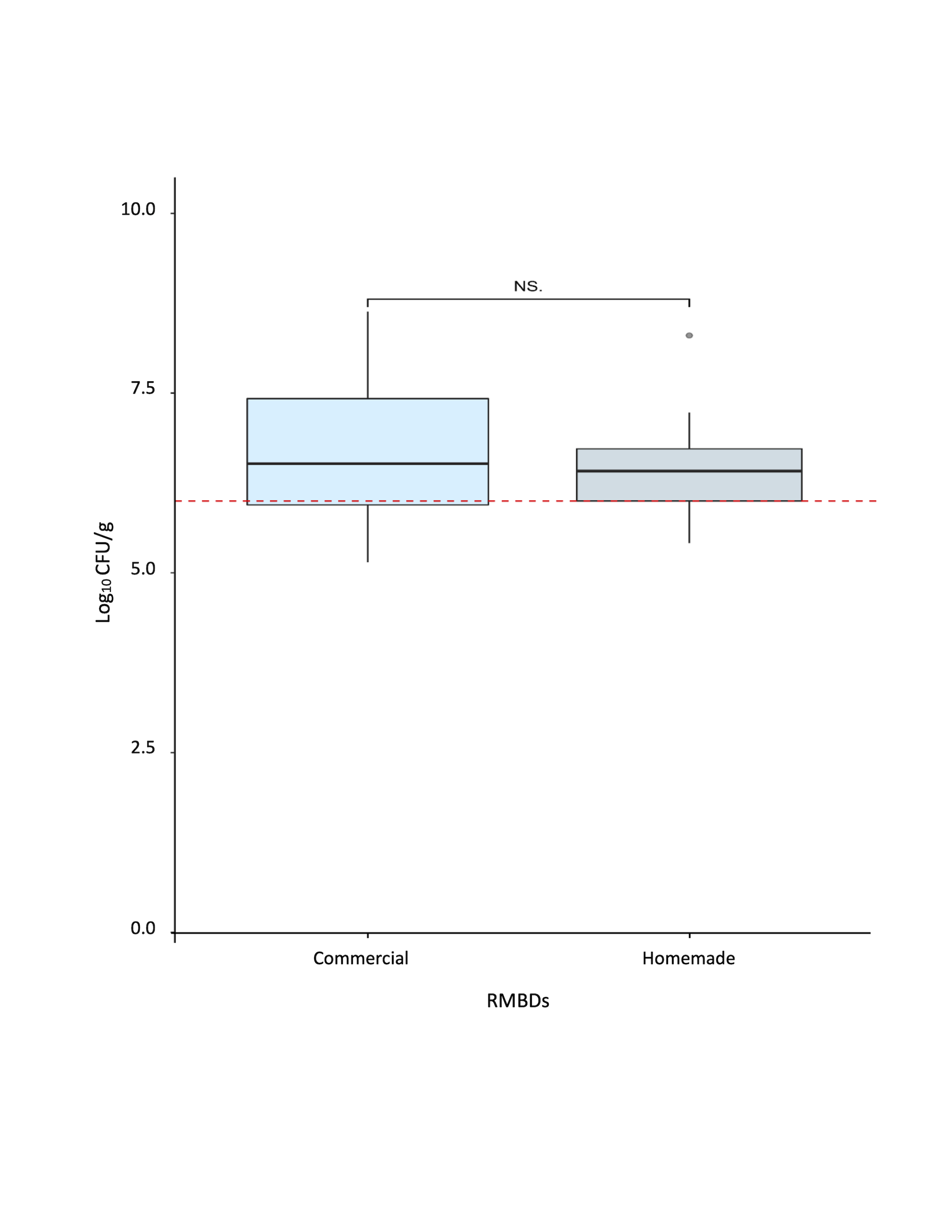

Supplement: Supplementary Figure 1 — Aerobic bacterial counts (APC) for commercial (n = 31) and homemade (n = 11) RMBDs. The Fligner-Killeen test was used to determine the homogeneity of variances and the Shapiro-Wilk test for normality. The level of APC between both diets was compared using Welch's Two-Sample T-test, p-value = 0.17. The red line shows the suggested APC upper limit for animal compound feed according to Kukier et al. (19). [file Image_1.TIFF]

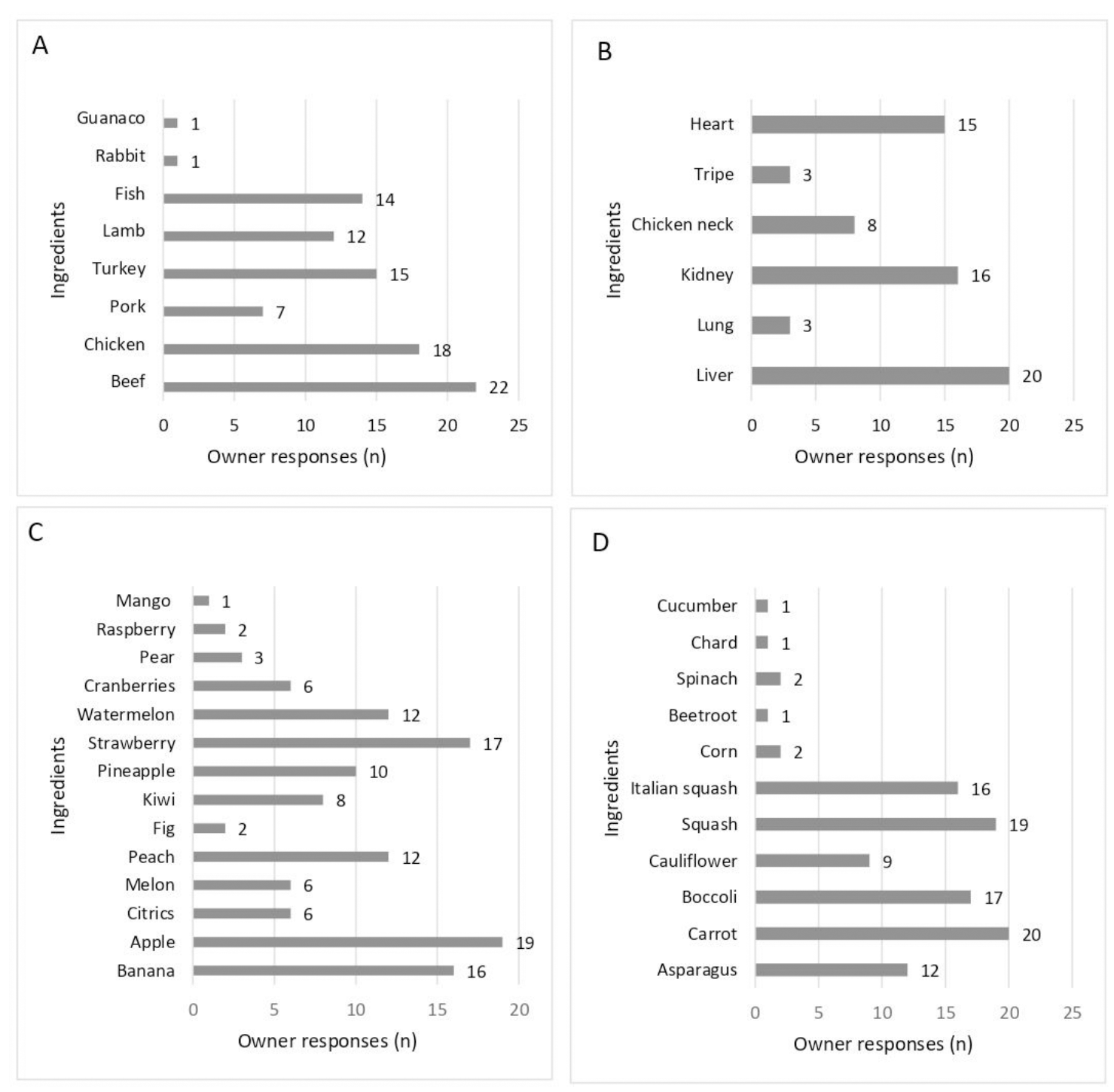

Supplement: Supplementary Figure 2 — Most common raw ingredients preferred by pet owners for commercial and/or homemade RBMDs (n = 42). (A) Meat, (B) Organ meats, (C) Fruits, (D) Vegetables. [file Image_2.TIFF]
